# The Effects of Copper Pollution on Fouling Assemblage Diversity: A Tropical-Temperate Comparison

**DOI:** 10.1371/journal.pone.0018026

**Published:** 2011-03-18

**Authors:** João Canning-Clode, Paul Fofonoff, Gerhardt F. Riedel, Mark Torchin, Gregory M. Ruiz

**Affiliations:** 1 Smithsonian Environmental Research Center, Edgewater, Maryland, United States of America; 2 CIMAR/CIIMAR – Centre of Marine and Environmental Research, Porto, Portugal; 3 Smithsonian Tropical Research Institute, Panama City, Republic of Panama; National Institute of Water & Atmospheric Research, New Zealand

## Abstract

**Background:**

The invasion of habitats by non-indigenous species (NIS) occurs at a global scale and can generate significant ecological, evolutionary, economic and social consequences. Estuarine and coastal ecosystems are particularly vulnerable to pollution from numerous sources due to years of human-induced degradation and shipping. Pollution is considered as a class of disturbance with anthropogenic roots and recent studies have concluded that high frequencies of disturbance may facilitate invasions by increasing the availability of resources.

**Methodology/Principal Findings:**

To examine the effects of heavy metal pollution as disturbance in shaping patterns of exotic versus native diversity in marine fouling communities we exposed fouling communities to different concentrations of copper in one temperate (Virginia) and one tropical (Panama) region. Diversity was categorized as total, native and non-indigenous and we also incorporated taxonomic and functional richness. Our findings indicate that total fouling diversity decreased with increasing copper pollution, whether taxonomic or functional diversity is considered. Both native and non-indigenous richness decreased with increasing copper concentrations at the tropical site whereas at the temperate site, non-indigenous richness was too low to detect any effect.

**Conclusions/Significance:**

Non-indigenous richness decreased with increasing metal concentrations, contradicting previous investigations that evaluate the influence of heavy metal pollution on diversity and invasibility of fouling assemblages. These results provide first insights on how the invasive species pool in a certain region may play a key role in the disturbance vs. non-indigenous diversity relationship.

## Introduction

A key question that has long puzzled ecologists is to understand which factors make ecosystems vulnerable to biological invasions [1], [2], [3]. Disturbance has been identified as a key factor in promoting invasions. Studies focused on the distribution of exotics in different systems have concluded that high frequencies of disturbance may facilitate invasions by increasing the availability of resources (e.g. space, light) and reducing competition with native species [4], [5], [6].

Estuaries and bays are an appropriate system to test the influence of disturbance on invasions, as these habitats are frequently exposed to an abundant supply of invasive larvae as a result of ballast water release, as well as to elevated regimes of anthropogenic disturbance. This makes fouling assemblages colonizing hard substrates in these environments extremely vulnerable to invasion [7], [8]. In this context, metal pollution is a typical pollutant within harbors and marinas, appearing in the form of antifouling paints, industrial waste and other sources [5], [9]. The most modern marine antifouling paints contain a copper based biocidal pigment and are applied to ship hulls and to several fixed structures (e.g. pilings, pontoons, buoys) to stop the growth of fouling organisms [10].

However and despite the efficiency of these copper-based coatings, fouling still occurs due to deteriorating paint, presence of biofilms, method of application, and increasing copper tolerances [11]. As a common pollutant in the marine environment, copper has been recognized as one of the three most toxic heavy metals to marine invertebrates, affecting their reproduction, growth, and abundance [9]. In addition, pollution can be considered a category of disturbance (anthropogenic) to an ecosystem and may affect community structure [12], [13]. Besides promoting invasion success by creating new habitats, introducing propagules and decreasing numbers of native species, these anthropogenic disturbances also deteriorate the capacity of the natives to resist new invaders [14].

Ballast tanks and ship hulls have been identified as major vectors for the transport and dispersal of nonindigenous species (NIS) [3], [15], [16] and research has shown that certain populations of NIS appear to have a superior tolerance to heavy metal pollution when compared to related native species [7], [17], [18], [19]. In a manipulative experiment aiming to test the effects of heavy metal pollution on the diversity and invasibility of marine hard-substrate communities in Australia, Piola and Johnston [5] found that increasing exposure to copper decreased native species diversity with no significant change in NIS. Copper exposure also increased the dominance (measured as percent cover in settling plates) of exotics [5]. Employing a different methodology in San Francisco Bay with fouling assemblages, Crooks et al. [20] recently showed a similar outcome: average native diversity was significantly sensitive to copper pollution while exotic richness was not. Both studies seem to confirm that anthropogenic shifts of abiotic determinants may facilitate the success and process of biological invasions and therefore, different repercussions at the level of pollution impacts and NIS management are expected [5], [20].

The probability of establishment of a non-native population and its expansion in a certain area/realm depends in part on the supply of potential invaders [3], [21]. In the marine realm, this so called ‘propagule pressure’ may change with the frequency of ship arrival [22]. Together with biotic and abiotic factors, this variation in propagule supply contributes to exotic diversity. However, exotic diversity should not be considered as a measure of invasibility by itself [2]. To account for the variation of propagule pressure in patterns of invasions, novel methods in propagule supply manipulation came to surface in recent years [22]. For example in a study developed in Australia, Clark and Johnston [23], successfully manipulated larvae of the invasive bryozoan *Bugula neritina* by injecting spawned larvae into containers with developing fouling communities. They explored the relationship between metal pollution and propagule supply and concluded that propagule pressure and disturbance interacted to affect fouling recruitment [23]. Another approach to account for propagule pressure is to experimentally manipulate environmental conditions (e.g. disturbance) using natural colonization [24]. Piola and Johnston [18] employed this method in marine fouling assemblages and concluded that the number of NIS increased with the exposure to metal pollution.

Most invasions in the marine system are described from temperate latitudes [25] but its probable causes remain relatively unexplored. However, several factors have been linked to such fact: (i) NIS follow the ‘*latitudinal gradient of species richness*’, which states that the tropics hold more species than do higher latitudes; (ii) more research attention or density of marine stations in temperate regions [3], [25]. In this context, there is no reason to presuppose that tropical marine communities are either more or less sensitive to copper and other heavy metal toxicants than temperate or boreal species. However, for individual species, at least in temperate environments, increased temperature often, but not uniformly, leads to increased toxicity. This may be as much a reflection of the increased metabolism of the organism and the speed with which it takes up the element, and more rapid damage than an intrinsic change to the means or mechanisms of toxicity. Alternatively, some species show a midrange optimum temperature at which toxicity is a minimum suggesting that these organisms are less affected by the toxin under otherwise less stressful conditions (see e.g., [26], [27]).

In addition, the importance of function has been recognized for the relationship between diversity and ecosystem functioning and sustainability [28], [29]. Functional differentiation based on relevant criteria better describes the ecological dissimilarity between species. As a result, the inclusion of this metric (whose parameters are detailed below) in biodiversity studies was proposed in recent studies (e.g., [30], [31], [32], [33], [34], [35]).

The present study examines the effects of metal pollution in exotic and native diversity in marine fouling communities. We conducted a field experiment in one temperate (Virginia) and one tropical (Panama) region, where species identity, functional identity and specific abundances (percent cover) were assessed. We hypothesize that (a) total diversity (taxonomic and functional) is sensitive to copper pollution (disturbance); (b) non-indigenous diversity (taxonomic and functional) is more tolerant to copper pollution than native diversity; (c) this scenario may differ across (tropic and temperate) regions.

## Materials and Methods

### Study sites and experimental design

The experiment lasted 9 weeks (September to December 2009) and was conducted, simultaneously, in two different biogeographic regions: Virginia's Eastern Shore Region (VA; 37°36′N, 75°41′W) and the Caribbean side of the Panama Canal, Panama (PA; 9°22′N, 79°57′W). At each region, we deployed 24 fibreglass plates (14×14×0.3 cm G-10 Epoxy glass). Plates were mounted on bricks using cable ties and suspended vertically on individual racks underneath docks at approximately 0.5 m depth.

**Figure 1:**
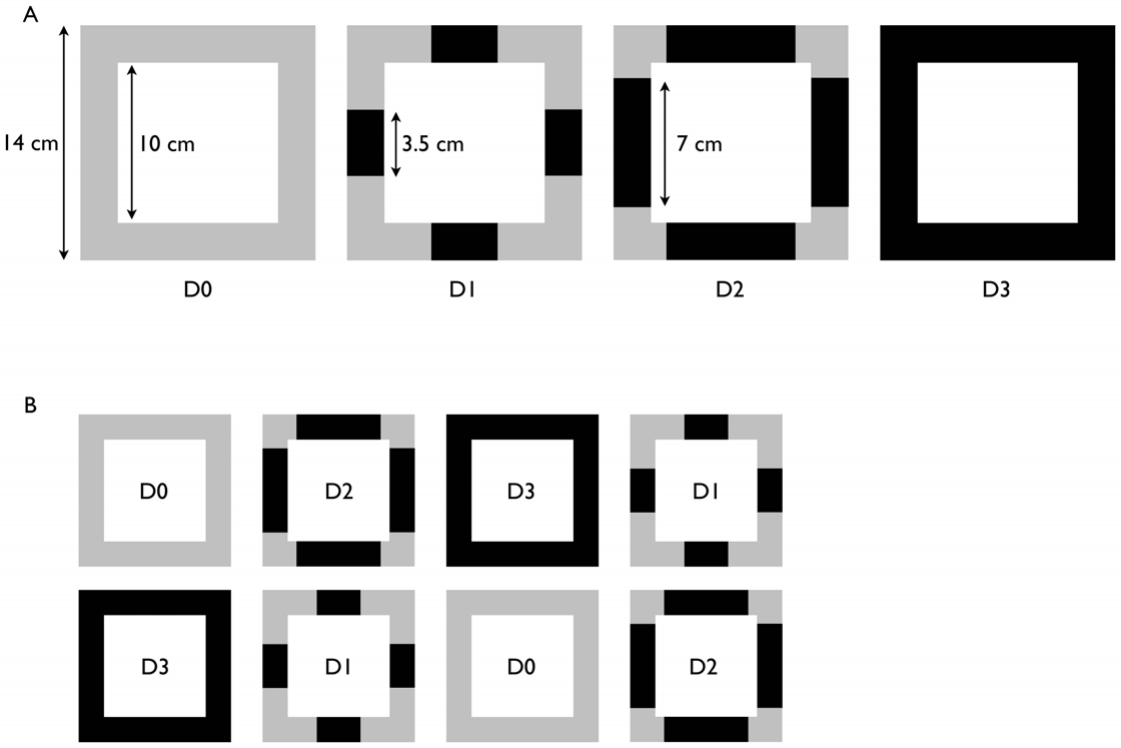
Diagram illustrating the experimental design employed. (A) We applied 4 different loads of a copper based antifouling paint: no disturbance (D0), 28 cm^2^ of AF paint (D1), 56 cm^2^ of AF paint (D2) and 96 cm^2^ of AF paint (D3). (B) Representation of one block with 2 replicates per treatment.

To test the effects of metal pollution on sessile invertebrate assemblages, we exposed these communities to different concentrations of copper. We applied different loads of the antifouling (AF) paint Interlux_®_ Ultra-Kote (76% Copper oxide) on the margins of a 100 cm^2^ colonization area in order to create a disturbance gradient: 96 cm^2^ of the non-toxic primer Primocon® (no disturbance or D0); 28 cm^2^ of AF paint and 68 cm^2^ of primer (disturbance 1 or D1); 56 cm^2^ of AF paint and 40 cm^2^ of primer (disturbance 2 or D2); and 96 cm^2^ of AF paint (disturbance 3 or D3) (Fig. 1A). In all treatments, 4 layers (each layer individually 75 microns thickness) of paint were applied.

We used a randomized block design to test for spatial heterogeneity with three blocks of 4 disturbance treatments. Each disturbance treatment was randomly replicated twice in each block resulting in 24 replicates per region (4 treatments×2 replicates×3 blocks = 24 plates) (Fig. 1B). Minimum distance between plates was 0.5 meters and minimum distance between blocks was 15 meters.

### Sampling and Functional Richness

After 9 weeks of colonization, all plates were retrieved from the field and photographed. For each plate we determined species richness, total cover and bare space by recording the number of species identified from the photographs using image analysis software CPCe [36]. Each image was sub-divided into a 3×3 grid of 9 cells, with 11 random points per cell resulting in 99 points analyzed per picture. This stratified random sampling method ensured that points were sampled in each region of the image [36]. In addition, each plate was carefully examined using a dissecting microscope to better measure total species pool. Sessile macroinvertebrates were identified to the lowest possible taxonomic group and assigned to four categories: native, NIS and cryptogenic (unspecified origin) based on existing literature reports, or to unresolved (based on an inability to identify to species level).

Functional groups (FG) encompass all species of a community which share a certain number of traits linked to ecological functions [37] and are typically defined according to the way in which they use and compete for any kind of resources (e.g. light, space) [30]. In this study, functional groups were determined according to five dimensions: body size, growth form, trophic type, modularity and motility (see Table 1 in [38], [39]). For each species, the functional group was defined as the set of ecological qualities realized at the adult stage. Here, we employed the following traits: body size (small, medium, large, very large), growth form (encrusting, massive, bushy, filamentous), trophic type (autotroph, suspension feeder, deposit feeder), modularity (solitary, colonial) and motility (attached), which could theoretically produce 4×4×3×2×1 = 96 functional groups.

**Table 1:** List of macroinvertebrates and their respective functional groups (see [38] for details) set by phylum found across the four disturbance treatments (D0–D3) in Panama (Pa) and Virginia (Vi) after 9 weeks of colonization.

Taxon	Functional	Site	Disturbance levels				Status	Source
	group		D0	D1	D2	D3		
Porifera								
*Chelonaplysilla erecta*	LESS	Pa	•	○	○	○	C	[57]
*Halichondria bowerbanki*	XMSS	Vi	••	••	••	○	C	[58]
*Halichondria melanadocia*	XMSS	Pa	••	•	○	••	N	[59]
*Haliclona tubifera*	XMSS	Pa	••	••	••	••	N	[59]
*Leucandra* sp.	LMSS	Pa	•	○	○	○	Unresolved	[59]
*Lissodendoryx spinulosa*	LMSS	Pa	○	•	○	○	N	
*Mycale arndti*	LESS	Pa	•	○	○	•	N	[59]
*Mycale microsigmatosa*	LESS	Pa	••	•	••	•	N	[59], [60]
*Sycon* sp.	LMSS	Pa	•	○	○	•	Unresolved	
*Tedania ignis*	XESS	Pa	•	•	•	•	N	[60]
Cnidaria								
*Edwardsia elegans*	LMSS	Vi	•	○	○	○	N	[61]
*Bougainvillia* sp.	LFSC	Pa	••	••	••	••	C	
*Cladonema radiatum*	LFSC	Pa	•	○	○	○	C	[59]
*Clytia* sp.	LFSC	Pa	•	•	•	•	Unresolved	
*Corydendrium parasiticum*	LBSC	Pa	•	○	○	○	C	[60]
*Eudendrium album*	LBSC	Vi	•	○	○	○	C	[62]
*Obelia bidentata*	MBSC	Vi	○	○	○	••	C	[62]
*Obelia bidentata*	MBSC	Pa	•	○	•	○	C	[60]
*Tubularia larynx*	LFSC	Vi	••	••	••	••	C	[62]
Unknown Anemone	LMSS	Vi	•	○	○	○	Unresolved	
Unknown Anemone	LMSS	Pa	○	•	○	○	Unresolved	
Bryozoa								
*Bugula neritina*	LBSC	Vi	•••	••	•••	••	NIS	[63]
*Bugula neritina*	LBSC	Pa	•	••	•	•	C	[64]
*Bugula stolonifera*	LBSC	Vi	••	••	••	○	N	[65]
*Aetea ligulata*	LBSC	Pa	○	○	•	•	C	[66]
*Anguinella palmata*	LBSC	Vi	••	•	•	•	C	[67]
*Bowerbankia* sp.	LBSC	Pa	•	○	○	○	Unresolved	
*Electra bengalensis*	LESC	Pa	••	••	•	○	NIS	[68]
*Savignyella lafontii*	LBSC	Pa	••	•	•	•	C	[60]
*Schizoporella pungens*	XESC	Pa	•	•	○	○	N	[60]
*Scrupocellaria carmabi*	LBSC	Pa	•	○	•	•	N	[66]
*Schizoporella* sp.	XESC	Vi	•	○	○	○	Unresolved	
Unidentified Bryozoan		Pa	•	○	○	○	Unresolved	
*Watersipora subtorquata*	XMSC	Pa	○	○	○	•	C	[64]
Chordata								
*Ascidia* sp.	LMSS	Pa	○	○	○	•	Unresolved	
*Diplosoma listerianum*	XESC	Pa	○	••	••	•	C	[69]
*Ecteinascidia turbinata*	LMSC	Vi	○	•	○	○	NIS	[70]
*Herdmania pallida*	LMSS	Pa	•	○	○	○	C	[71]
*Molgula manhattensis*	LMSS	Vi	•	••	••	••	N	[72]
*Perophora viridis*		Pa	•	○	○	○	N	[72]
*Phallusia nigra*	LMSS	Pa	○	•	○	○	NIS	[69]
*Styela canopus*	LMSS	Pa	••	•	•	•	NIS	[69]
*Symplegma brakenhielmi*	XESC	Pa	•••	•••	•••	•	C	[73]
Crustacea								
*Amphibalanus improvisus*	MMSS	Vi	•••	•••	•••	•••	N	[74]
*Amphibalanus improvisus*	MMSS	Pa	••	••	••	••	N	[74]
*Corophium* sp.	MMSS	Vi	••	••	••	••	Unresolved	
Polychaea								
*Polydora cornuta*	LFSS	Vi	○	○	•	•	N	[75]
*Branchiomma bairdi*	LMSS	Pa	••	••	••	••	N	[76]
*Hydroides elegans*	LMSS	Pa	••	••	••	••	NIS	[77]
*Pileolaria militaris*	MMSS	Pa	••	••	••	••	C	[78]
*Pomatoceros minutus*	MMSS	Pa	••	•	•	•	C	[79]
*Salmacina tribranchiata*	XMSC	Pa	••	••	••	••	N	[79]
*Spirorbis* sp.	MMSS	Vi	•	○	○	○	Unresolved	
*Spirorbis tuberculatus*	MMSS	Pa	••	••	••	••	NIS	[80]
Mollusca								
*Anomia peruviana*	LMSS	Pa	••	••	••	••	NIS	Canning-Clode, unpublished
Ostreidae	LMSS	Pa	•	•	•	•	Unresolved	

Appearance of organisms is shown by ○, not present; •, ≤1% mean cover; ••, <10% mean cover; •••, >10% mean cover; Taxa were also classified as native (N), non-indigenous (NIS) and cryptogenic (C) based on literature, or to unresolved (based on an inability to identify to species level).

### Copper content analysis

Water samples were taken twice after 3 and 6 weeks in Virginia to test Cu leaching from the AF paint. Eight plates from one block (2 replicates per disturbance treatment) were placed individually in buckets with 2L of seawater for a 2 h period. Each bucket was aerated to provide O_2_ and to ensure water mixing. A volume of 50 ml of seawater per treatment (n = 2) was then filtered to a polypropylene sample tubes using a syringe and disposable syringe filters (Whatman* GD/X 25 mm). To prevent contamination nitrile gloves were used during this procedure. Water samples were kept refrigerated, brought to the laboratory as soon as possible, and acidified to 0.5% V/V with ultrapure HNO_3_. Cu content was determined within 3 months after sampling. Water samples were extracted with APDC-NaDDDC/chloroform and diluted into 6% ultrapure HNO_3_ to remove the seawater matrix and concentrate the samples following the methods of Riedel et al. [40]. The samples were analyzed for Cu by inductively coupled plasma-mass spectrometry (ICP-MS) using a Perkin-Elmer Elan II. These eight plates were brought back to the field within 3 hours of each sampling event but were not considered for the community structure analysis.

To test whether the biota present in the colonization area of each treatment was accumulating copper we analyzed the tissue of the most common organism across all treatments in Virginia (the barnacle *Amphibalanus improvisus*). At the end of the experiment four individuals of *Amphibalanus improvisus* per treatment were sampled whenever possible from the central area of the plate. Samples of dry tissue were digested with ultrapure HNO_3_, HCl and HClO_4_ in open Teflon® vials, and diluted with 0.5% ultrapure HNO_3_ for Cu analysis by ICP-MS, following the methods of Riedel and Valette-Silver [41].

### Statistical analysis

A one-way ANOVA was performed to test Cu leaching from the AF paint after 3 and 6 weeks. A one-way ANOVA was also used to test the copper accumulation from the barnacle *Amphibalanus improvisus* across disturbance. In case of a significant effect, the Tukey's HSD *post hoc* analysis identified which paint dosages differed in their efficiency in leaching and causing accumulating of copper in organisms on the panels.

Hypotheses about the effects of disturbance, block and their interaction in species and functional richness of fouling assemblages were tested with two separate two-factorial ANOVA for each region. Blocks were treated as a random factor (3 levels) and disturbance as a fixed factor (4 levels). Diversity measures (dependent variables) included total richness (taxonomic and functional), native richness (taxonomic and functional), invasive richness (taxonomic and functional), and cryptogenic richness. Homogeneity of variances was tested with the Cochran's test and dependent variables were Log_10_ transformed if needed. Tukey's HSD *post hoc* analysis was used to examine significant effects of disturbance in diversity.

For multivariate analysis, taxonomic and functional richness at both regions were contrasted across disturbance treatments and blocks using a two-factor permutational multivariate ANOVA (PERMANOVA) where disturbance was operated as a fixed factor and block as random factor. We used the SIMPER routine to measure the contribution of each taxon to average dissimilarities between controls and the highest disturbed treatment. The more significant taxa causing these dissimilarities were identified [42]. SIMPER and PERMANOVA analysis were performed with PRIMER 6 [43] and its PERMANOVA+add-on [44].

## Results

After 9 weeks of colonization, in Virginia we found 16 macroinvertebrates and 9 FG and Panama's plates were colonized by 40 species and 12 FG (Table 1). In Virginia, 5 species were identified as native (31%), 2 as NIS (13%) and 6 as cryptogenic (38%). Plates from Panama included 12 natives (30%), 14 cryptogenic (35%) and 6 NIS, (15%). Barnacles and hydroids were more abundant in Virginia whereas numbers of sponges and tunicates were higher in Panama. In addition, barnacles appear to be more tolerant to copper pollution as their average abundance does not change with increasing disturbance (Table 1).

**Figure 2:**
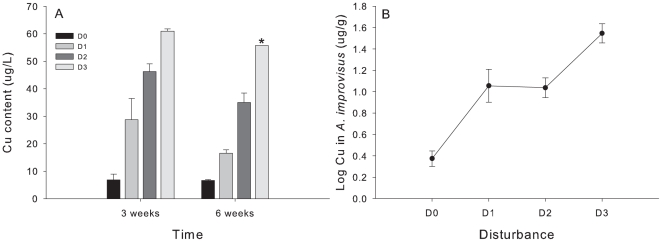
Test for treatment's efficiency. (A) Copper content from water samples after a 2 hr exposure to disturbance panels taken after 3 and 6 weeks in Virginia from independent buckets containing individual disturbance treatments (n = 2); (B) Quantity of copper measured after 9 weeks from the tissue of *Amphibalanus improvisus*, the most common organism across all disturbance treatments in Virginia (n = 4). Means and standard deviations are indicated. Disturbance treatments abbreviations are as in Figure 1. * There is no standard deviation at D3 after 6 weeks as there was only one replicate.

The applied disturbance treatments were effective as shown in Figures 2, S1 and S2. Figures S1 and S2 show examples of individual fouling communities across disturbance treatments in both study sites. After 3 and 6 weeks in Virginia, average concentration of copper significantly increased with the different dosages of AF paint (ANOVA - 3 weeks: F = 59.59, P<0.01; 6 weeks: F = 94.36, P<0.01). D3 was not included in the 6 weeks model due to the loss of replicates. *Post hoc* analysis revealed that copper dosages were all significantly different from each other (Fig. 2A; Tukey's HSD<0.05). Although mean copper concentration from the different treatment seems to decrease in time, no significant differences were found. In addition, at the end of the experiment, the accumulation of copper in *Amphibalanus improvisus* significantly increased with disturbance (ANOVA: F = 20.48, P<0.01). With the exception of D1 and D2, *post hoc* analysis determined that copper concentration in barnacles were significantly different across disturbance treatments (Fig. 2B; Tukey's HSD<0.05).

**Figure 3:**
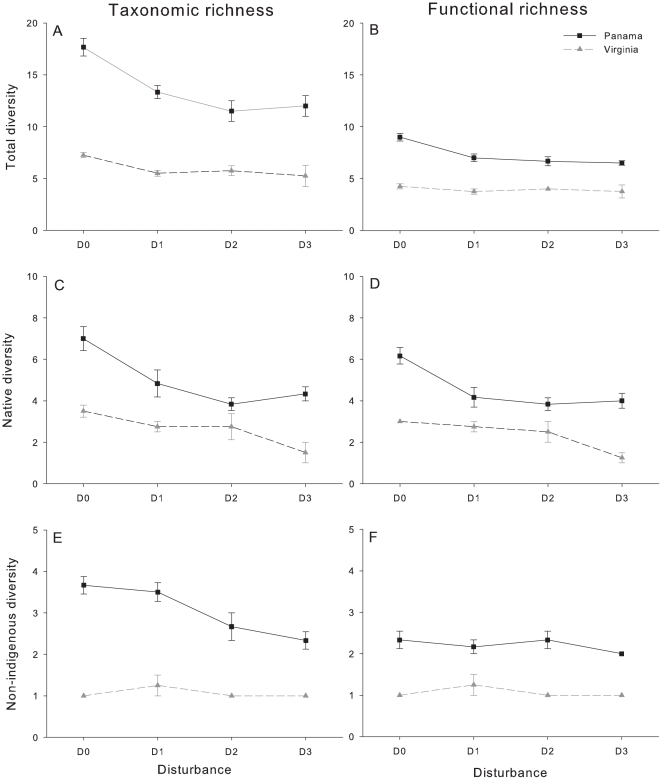
Relationship between disturbance and different measures of diversity in Virginia and Panama. Diversity measures are: total taxonomic richness (A); total functional richness (B); native taxonomic richness (C); native functional richness (D); non-indigenous taxonomic richness (E); and non-indigenous functional richness (F). Means and standard errors are indicated (n = 4 in Virginia; n = 6 in Panama). Disturbance treatments abbreviations are as in Figure 1.

The two-factorial ANOVA performed for each region did not detect any block effect, which indicates that the experimental units were heterogeneously distributed (Table 2). In general, diversity was sensitive to increasing copper exposure in Panama while in Virginia only native functional richness was affected by copper disturbance (Fig. 3; Table 2). In Panama, total number of species and FG significantly decreased with disturbance where *post hoc* testing identified (Tukey's HSD<0.05) differences between the controls and the disturbance treatments (Fig. 3A–B). No differences among the disturbed plates were detected. Similarly, Panama's native diversity (taxonomic and functional) was affected by disturbance (Fig. 3C–D; Table 2). More species and FG were observed in the untreated plates (Tukey's HSD<0.05). In Virginia, *post hoc* analysis identified significant differences in native functional diversity between D0 and D3 and between D1 and D3 (Fig. 3D). No significant relationship between non-indigenous diversity and disturbance was observed in Virginia likely because the invasive signal was too low (Fig. 3E–F). In contrast, Non-indigenous species in Panama were sensitive to disturbance with significantly more NIS in the controls and D1 than in D3 (Fig. 3E; Table 2).

**Table 2:** Results from the 2-factorial ANOVA on different diversity measures for Virginia and Panama.

		Virginia				Panama			
Diversity measure	Source of	df	MS	F	P - value	df	MS	F	P - value
	variation								
Total species richness	D	3	3.23	5.74	0.092	**3**	**47.15**	**9.15**	**0.011**
	B	1	0.06	0.11	0.761	2	3.88	0.75	0.511
	D*B	3	0.56	0.29	0.831	6	5.15	1.16	0.390
	Error	8	1.94			12	4.46		
Total functional richness*	D	3	0.23	3.67	0.157	**3**	**0.02**	**5.56**	**0.036**
	B	1	0.56	9.00	0.058	2	0.00	0.06	0.946
	D*B	3	0.06	0.09	0.963	6	0.00	1.66	0.215
	Error	8	0.69			12	0.00		
Native taxonomic richness*	D	3	2.75	6.60	0.078	**3**	**0.08**	**10.20**	**0.009**
	B	1	2.25	5.40	0.103	2	0.01	1.35	0.329
	D*B	3	0.42	0.56	0.659	6	0.01	0.54	0.766
	Error	8	0.75			12	0.01		
Native functional richness	D	**3**	**2.42**	**14.50**	**0.027**	**3**	**7.15**	**7.25**	**0.020**
	B	1	1.00	6.00	0.092	2	0.54	0.55	0.604
	D*B	3	0.17	0.44	0.728	6	0.99	1.03	0.453
	Error	8	0.38			12	0.96		
Invasive taxonomic richness	D	3	-	-	-	**3**	**2.49**	**7.78**	**0.017**
	B	1	-	-	-	2	0.04	0.13	0.880
	D*B	3	-	-	-	6	0.32	0.70	0.657
	Error	8	-			12	0.46		
Invasive functional richness	D	3	-	-	-	3	0.15	1.00	0.455
	B	1	-	-	-	2	0.04	0.27	0.770
	D*B	3	-	-	-	6	0.15	0.73	0.633
	Error	8	-			12	0.21		
Cryptogenic richness	D	3	4.06	4.53	0.123	3	5.50	2.40	0.166
	B	1	0.06	0.07	0.809	2	0.79	0.35	0.721
	D*B	3	0.90	0.75	0.550	6	2.29	1.53	0.250
	Error	8	1.19			12	1.50		

*Data was log_10_ transformed for total functional richness and native taxonomic richness in PA. Analysis was not performed for non-indigenous richness in Virginia due to a weak signal (only two species: *Bugula neritina* present in all disturbance treatments and *Ecteinascidia turbinata* in only one treatment). Significant results (P<0.05) highlighted in bold (n = 4 in Virginia; n = 6 in Panama). Disturbance = D and Block = B represent the source of variation.

No significant relationship between disturbance and total diversity was observed in Virginia (although there is a marginal significance for total species richness – Table 3). In addition, native diversity (in terms of both taxonomic and functional) and cryptogenic species are negatively affected by disturbance in Virginia. In Panama, metal pollution significantly reduces total and native diversity. Furthermore, numbers of NIS also significantly decreased with enhancing copper pollution in Panama (Table 3). The available space on the settling plates was also affected by disturbance in Panama as average open space increased with disturbance (D0: 26.6%±16.2; D1: 35.5%±11.9; D2: 61.3%±19.3; D3: 55.6%±22.9). In Virginia, open space was constant across disturbance treatments (average open space between 60 and 45%).

**Table 3:** Effects of disturbance (independent variable) on diversity (dependent variable) of fouling communities.

Diversity measure	Virginia		Panama	
	R^2^	*P*-value	R^2^	*P*-value
Total species richness	0.25	0.051	**0.46**	**0.000**
Total functional richness*	0.05	0.430	**0.43**	**0.001**
Native taxonomic richness*	**0.40**	**0.008**	**0.33**	**0.003**
Native functional richness	**0.51**	**0.001**	**0.35**	**0.002**
Invasive taxonomic richness	0.01	0.670	**0.47**	**0.000**
Invasive functional richness	0.01	0.670	0.05	0.281
Cryptogenic richness	**0.32**	**0.022**	0.15	0.061

Results of the linear regression analysis are shown for Virginia and Panama.

*Data was log_10_ transformed for total functional richness and native taxonomic richness in PA. Significant results (P<0.05) highlighted in bold (n = 4 in Virginia; n = 6 in Panama).

We performed separate multivariate analysis on the effects of disturbance on community composition at each region and found that significant differences at both regions were observed between disturbance treatments (Table 4). In addition, PERMANOVA detected a block effect in community composition in Virginia, which probably reflects a lower replication at this region. According to SIMPER routines, three species and three FG were essential in differentiating control from D3 assemblages in Virginia. Average abundance of the barnacle *Amphibalanus improvisus* increased with disturbance while a higher abundance of the exotic *Bugula neritina* was found in the controls (Table 5). Accordingly, the exotic *Anomia peruviana* also had a 9% negative contribution to dissimilarities between treatments in Panama, while the abundances of two native species increased with disturbance.

**Table 4:** Summary of the two-factor PERMANOVA of the multivariate data.

Site	Source of	Taxonomic richness				Functional richness			
	variation	df	MS	Pseudo-*F*	*P*-value	df	MS	Pseudo-*F*	*P*-value
Virginia	B	**1**	**2431.10**	**3.05**	**0.030**	**1**	**2495.60**	**4.11**	**0.025**
	D	3	1741.10	2.54	0.150	**3**	**1572.50**	**4.87**	**0.049**
	BxD	3	685.64	0.86	0.601	3	322.74	0.53	0.824
	Residual	8	795.98			8	607.24		
	Total	15				15			
Panama	B	2	2225.80	1.22	0.2869	2	1234.10	0.94	0.510
	D	**3**	**3572.00**	**2.35**	**0.0185**	**3**	**2872.00**	**2.24**	**0.042**
	BxD	6	1521.40	0.83	0.7576	6	1282.30	0.97	0.514
	Residual	12	1822.20			12	1317.90		
	Total	23				23			

Significant results (P<0.05) highlighted in bold (n = 4 in Virginia; n = 6 in Panama). Disturbance = D and Block = B represent the source of variation.

**Table 5:** Results from the SIMPER routine performed with multivariate data from both Panama and Virginia to identify which species or FG contributed more (≥10%) to observed changes in community composition between untreated controls (D0) and highest disturbance (D3).

	Taxonomic diversity			Functional diversity	
Site	Source	Status	Contribution (%)	Source	Contribution (%)
Virginia					
	*Amphibalanus improvisus*	N	39(+)	MMSS	44(+)
	*Bugula neritina*	NIS	25(−)	LBSC	32(−)
	*Tubularia larynx*	C	12(+)	LFSC	12(+)
Panama					
	*Symplegma brakenhielmi*	C	25(−)	XESC	34(−)
	*Salmacina tribranchiata*	N	10(+)	LMSS	15(−)
	*Haliclona tubifera*	N	10(+)	XMSS	14(−)
	*Anomia peruviana*	NIS	10(−)	XMSC	12(+)

Taxa classified as native (N), non-indigenous (NIS) and cryptogenic (C) based on literature. Contribution (%) and direction of change (+ positive; - negative) are indicated (n = 4 in Virginia; n = 6 in Panama).

## Discussion

In this study, we examined the effects of copper pollution (disturbance) on diversity of fouling assemblages in a temperate and a tropical region using an expanded approach: diversity was categorized as total, native and non-indigenous and we also incorporated taxonomic and functional richness. Moreover, to the best of our knowledge, this is the first study to directly compare the response of tropical and temperate fouling assemblages to copper exposure. Our findings indicate that total fouling diversity is sensitive to metal pollution, whether taxonomic or functional diversity is considered. Thus, the shape of the relationship between disturbance and total diversity is more pronounced in the tropics. Similarly, disturbance also played a key role in decreasing native diversity and non-indigenous species richness in Panama. In fact, tropical assemblages appear to be more sensitive to copper exposure relative to temperate assemblages probably because increased temperature often leads to increased toxicity (see eg., [26], [27]).

One factor that has frequently been suggested to control biodiversity in different systems is disturbance [45], [46]. However, a universal definition of disturbance is debatable, as its classification ranges from abiotic to biotic or natural to anthropogenic [47], [48]. Disturbance has been often defined as the loss of biomass [49] which can facilitate the establishment of new individuals by altering the resource opportunities available to the species in a system [50], [51]. Disturbance has also been defined as an ‘ecological disruption that leads to some type of open opportunity or vacant area in a community [13]. We believe we have created a disturbance regime by applying different loads of an antifouling (AF) paint composed of a heavy metal toxicant (Cu) in the margins of settling plates. Thus, with samples taken from water as well as from the most abundant organism across all treatments, we demonstrated that the applied disturbance treatments were effective. We showed for two periods in time (3 and 6 weeks) a clear increasing pattern between the concentration of copper taken from water samples and the different dosages of AF paint. This indicates that the pollutant (Cu) was leached from the AF paint in different concentrations creating a clear disturbance gradient. Additionally, we also demonstrate that the barnacle *Amphibalanus improvisus* has accumulated copper with increasing disturbance implying that the biota colonizing the area delimited by the AF paint in the different treated plates has accumulated distinctive copper concentrations.

It is widely considered that disturbance can have variable effects on diversity causing a variety of shapes between the two factors [47], [48], [52]. One conceptual formulation of the effects of disturbance on diversity is the intermediate disturbance hypothesis (IDH, [45]) that predicts a unimodal relationship with maximum diversity at ‘intermediate’ levels of disturbance. The foundation behind this concept is that high frequencies of disturbance and longer-lived species cannot persist in the same system; at low disturbance strong competitors force pioneer species to extinction; at intermediate rates of disturbance, diversity is maximized due to the coexistence of competitors and colonizers [45]. However, a recent meta-analytical comparison examining 94 studies on the diversity–disturbance relationship in different systems has shown that the unimodal pattern was only observed in 18% of the studies [52]. In their review, Hughes *et al*. [52] found that disturbance most commonly decreases diversity. Although our experiment was too short for an adequate test of the IDH, we also found that disturbance significantly decreased total species richness in both sites (it is marginally significant in Virginia probably due to lower replication – see table 3) and total functional richness in Panama.

In the present study, we have demonstrated that numbers of native species (and FG) are strongly reduced with augmenting the concentration of copper. This seems to be in consensus with recent investigations that used copper as a disturbance in fouling assemblages [5], [20]. Piola and Johnston [5] performed a manipulative experiment in Australia to evaluate the influence of heavy metal pollution on diversity and invasibility of marine hard-bottom assemblages. In order to create an increasing pollution regime, they also used coatings of a copper-based antifouling agent. Their findings indicate that by increasing pollution exposure, native species diversity was severely reduced [5]. More recently, Crooks *et al*. [20] conducted an experiment in San Francisco Bay to investigate the role of abiotic factors in affecting the invasibility of a community. In their study, PVC plates were periodically removed from the field and placed into buckets with different copper concentrations for a 72 h period before being returned to the Bay. Although a different experimental design was employed, Crooks *et al*. [20] concluded that average native species richness was significantly reduced by copper exposure, as the present study.

In this study, the average number of NIS in Panama significantly decreases with augmenting copper concentration, which partially contrasts the findings of the two previously mentioned studies [5], [20]. Piola and Johnston have not found a significant change in non-indigenous richness with increasing copper exposure but concluded that the spatial dominance of NIS (measured as percentage cover) increased with metal pollution in all their study sites [5]. Similarly, Crooks *et al*. 's study concluded that their exotic species pool was not sensitive to copper exposure [20]. The absence of any significant pattern for NIS in Virginia is probably due to a weaker invasive signal (only 2 NIS were found) when compared to Panama. Native diversity at both sites displayed similar patterns with disturbance (linear negative relationship) as they show similar native signals (33% for Panama and 31% for Virginia). However, our observation that non-indigenous richness was higher in the tropics seems to be consistent with recent reviews that regard NIS to follow the latitudinal gradient of species richness, with diversity decreasing towards the poles [3], [25]. Higher numbers of NIS in Panama were expected, as our study site was located in the eastern mouth of the Panama Canal, considered a key vector in promoting biological invasions [53].

In addition, this observed invasion pattern across latitude has also been linked to other factors such as historical baseline information, propagule supply, resistance to invasion and disturbance [25]. Furthermore, recent studies showed that species rich or poor communities located in tropical waters are more susceptible to invasions [54], [55]. However, although we found more NIS in the tropics, it should be noted at this point that this study did not cover intermediate regions between Panama and Virginia. Having more study sites across latitude would be beneficial to support the idea that NIS are following the latitudinal gradient of species richness. Moreover, there was a large percentage of species in both systems that could not be resolved as ‘native’ or ‘NIS’ (these were categorized as cryptogenic or unresolved). However, because total diversity (where all cryptogenic species were included) decreased with copper exposure in both regions, we believe that this lack of resolution would likely not impact conclusions concerning the role of disturbance to native or NIS diversity.

We conclude that diversity is sensitive to copper pollution in fouling assemblages, whether taxonomic or functional richness is considered. Native diversity was severely reduced by disturbance in both sites, and more importantly, non-indigenous richness decreased with increasing metal concentrations, contradicting previous investigations. This pattern only occurred in the tropics most likely due to the different proportions of NIS per site (more NIS in the tropics). This study also corroborates that pollution is a category of disturbance (anthropogenic) as we show it affects total diversity and availability of resources (open space). Finally, this investigation represents the first study exploring the effects of metal pollution on diversity that incorporates functional diversity in addition to species richness as a dimension of biodiversity. Functional diversity was consistently less sensitive to copper pollution than species richness possibly because toxicant sensitivities are considered to be highly species specific and substitution within functional groups may obscure structural impacts on communities. This corroborates recent studies that confirmed species richness as the most sensitive indicator of pollution effects on biodiversity [56].

## Supporting Information

Figure S1 **Individual replicates from fouling communities in Virginia across disturbance treatments.** Panel A – D0; panel B – D1; panel C – D2 and panel D – D3. See Methods for details.
(TIF)

Figure S2 **Individual replicates from fouling communities in Panama across disturbance treatments.** Panel A – D0; panel B – D1; panel C – D2 and panel D – D3. See Methods for details.
(TIF)
