# Supplementary figures and images for: The Effects of Copper Pollution on Fouling Assemblage Diversity: A Tropical-Temperate Comparison
Source: PLoS One. 2011 Mar 18;6(3):e18026. doi: 10.1371/journal.pone.0018026 (PMC3060921; doi:10.1371/journal.pone.0018026)

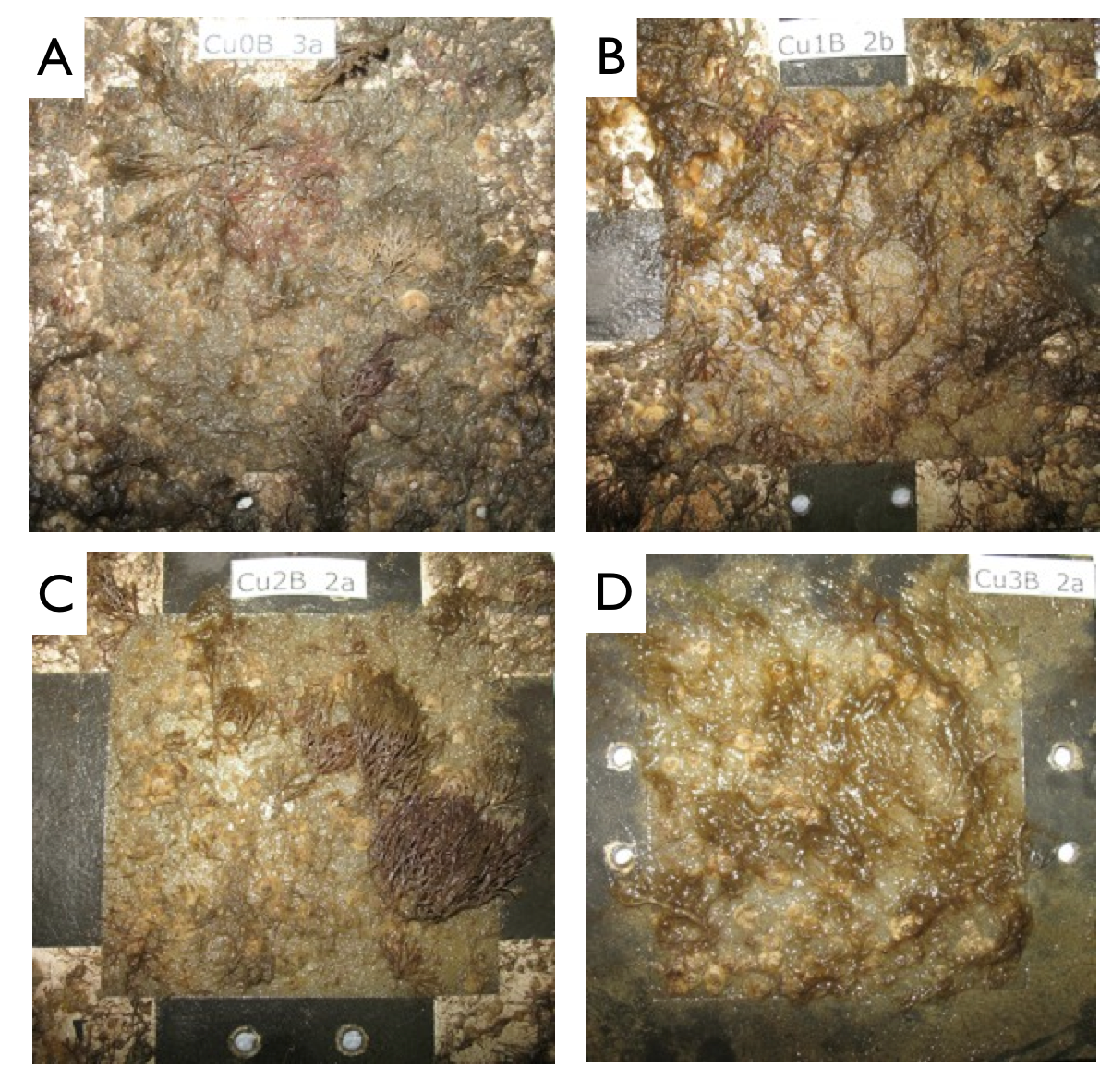

Supplement: Figure S1 — Individual replicates from fouling communities in Virginia across disturbance treatments. Panel A – D0; panel B – D1; panel C – D2 and panel D – D3. See Methods for details. (TIF) [file pone.0018026.s001.tif]

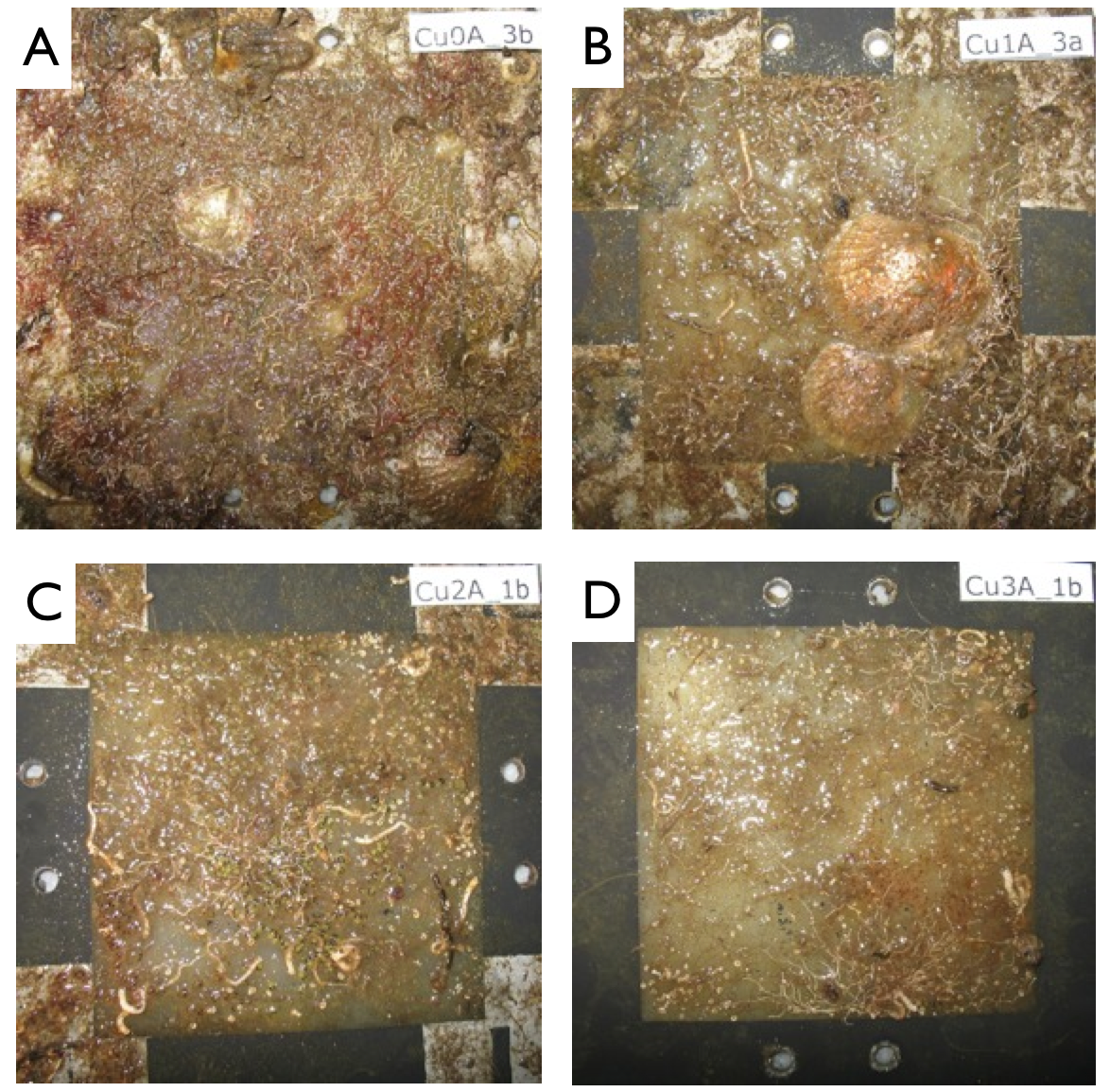

Supplement: Figure S2 — Individual replicates from fouling communities in Panama across disturbance treatments. Panel A – D0; panel B – D1; panel C – D2 and panel D – D3. See Methods for details. (TIF) [file pone.0018026.s002.tif]
